# [^68^Ga] Ga-FAPI positron emission tomography/computed tomography uptake following liver stereotactic body radiation therapy in hepatocellular carcinoma: two case reports

**DOI:** 10.1186/s41824-026-00315-6

**Published:** 2026-11-09

**Authors:** M. Shivacharan, Sumeet Suresh Malapure, Kirthan Chatra, Shirley Lewis

**Affiliations:** 1 Department of Radiation Oncology, Kasturba Medical College, Manipal Academy of Higher Education, Manipal, India; 2 Department of Nuclear Medicine, Kasturba Medical College, Manipal Academy of Higher Education, Manipal, India; 3 Department of Radiodiagnosis and Imaging, Kasturba Medical College, Manipal Academy of Higher Education, Manipal, India

**Keywords:** Hepatocellular carcinoma, SBRT, [^68^Ga]Ga-FAPI PET/CT, Radiation fibrosis, False-positive, Tumour microenvironment, Multimodal imaging, LI-RADS, Cancer-associated fibroblasts, Liver imaging

## Abstract

**Background:**

[⁶⁸Ga]Ga-FAPI Positron Emission Tomography/Computed Tomography have shown high sensitivity for detecting hepatocellular carcinoma (HCC). [⁶⁸Ga]Ga-FAPI uptake also occurs in benign fibroinflammatory processes, creating a specific challenge for post-treatment surveillance after liver-directed therapies.

**Case presentation:**

We report two HCC patients who underwent stereotactic body radiation therapy (SBRT) and subsequently developed FAPI-avid hepatic lesions. In Case 1, a 68-year-old man showed focal FAPI uptake in segment VI following SBRT, which Magnetic Resonance Imaging (MRI) characterised as fibrosis (LI-RADS LR-TR non-viable), with stable tumour markers and no clinical progression. In Case 2, a 77-year-old man exhibited FAPI avidity in the treated lesion, with MRI findings suggestive of post-treatment change. In both cases, the increased FAPI uptake was deemed as false-positive and attributed to post-radiation fibroinflammatory changes.

**Conclusion:**

These cases highlight a critical interpretative limitation of [⁶⁸Ga]Ga-FAPI PET/CT in post-SBRT HCC surveillance. Fibroblast activation in healing tissues can mimic recurrence; MRI correlation should be done to prevent misdiagnosis. Our report provides novel evidence that SBRT-induced hepatic fibrosis may confound FAPI-based imaging and adds to the evolving understanding of FAPI’s performance in surveillance of liver malignancies.

## Introduction

Stereotactic body radiation therapy (SBRT) is an established, non-invasive treatment for hepatocellular carcinoma (HCC) (Navin et al. 2022). The emergence of [^68^Ga]Gallium-fibroblast activation protein inhibitor (FAPI) PET/CT, a molecular imaging technique targeting cancer-associated fibroblasts, has shown great promise for HCC detection, often outperforming traditional [^18^F]FDG PET/CT. However, the very mechanism that makes FAPI PET/CT effective, its high affinity for activated fibroblasts, also makes it susceptible to misinterpretation in the post-SBRT setting, where radiation-induced tissue injury stimulates significant fibroblast activity (Sangro et al. 2025; Serafini et al. 2022) Post-SBRT fibroblast activation may result in FAPI uptake without a viable tumour, making MRI correlation important for accurate assessment (Yip et al. 2020; Yarnold and Vozenin Brotons 2010). We present two cases of HCC post-SBRT imaged with [⁶⁸Ga]Ga-FAPI PET/CT that address this clinical dilemma.

## Case summary

### Case 1

A 68-year-old gentleman with biopsy-proven (lytic left-iliac-crest lesion (AFP 712 ng/mL, Child-Pugh A)) oligometastatic HCC, alcohol-related liver disease, type 2 diabetes mellitus, and hypertension, presented for routine post-SBRT surveillance. Baseline [⁶⁸Ga]Ga-FAPI PET/CT revealed two hepatic lesions (segments V/VI and IV) with bone metastasis. His treatment history included SBRT to liver (50 Gy/5# completed November 11, 2024) and left iliac crest (30 Gy/3# completed February 2024), along with over 25 cycles of systemic Atezolizumab + Bevacizumab therapy.

#### Clinical course and diagnostic assessment

**Fig. 1:**
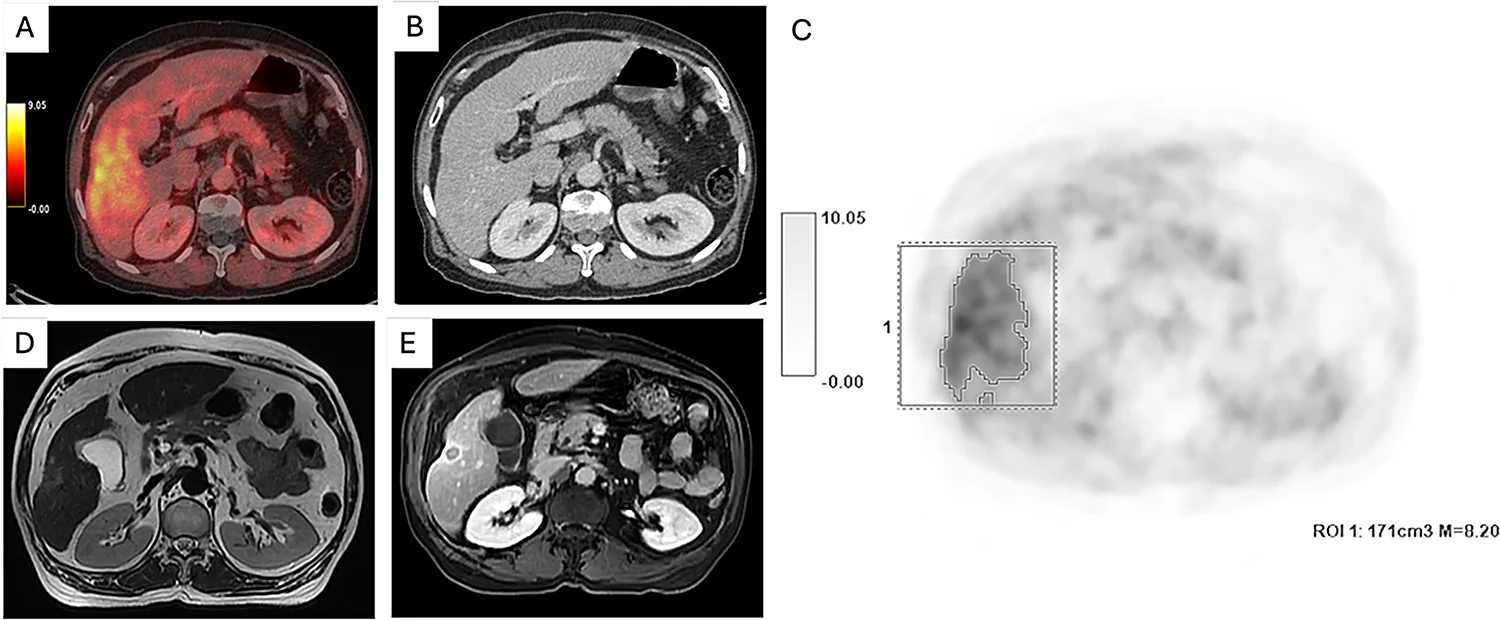
(**A**) Fused Gallium 68 Fibroblast activated protein inhibitor (Ga-68 FAPI) Positron Emission Tomography/Contrast enhanced computed tomography (PET/CECT) image showing diffusely increased FAPI uptake in segment V and VI of liver with standardised uptake value maximum of 8.20. (**B**) The corresponding axial section of the CECT image shows no significant hypodense lesion corresponding to the lesion on CECT. The MRI shows well-defined T2 hypointense (**C**) Standalone PET image in grayscale bar showing the maximum standardised uptake value(M = 8.20). (**D**) T1 hyperintense lesion (**E**) showing mild capsular retraction seen in segment V of liver with adjacent smooth peripheral enhancing area not showing washout

The patient’s clinical course is summarised in Table 1. In June 2025, a [⁶⁸Ga]Ga-FAPI PET/CT scan showed an interval increase in FAPI avidity in the previously SBRT-treated liver segment VI. This finding was initially concerning for tumour recurrence. However, a concurrently performed MRI of the abdomen (June 24, 2025) revealed a T1 hyperintense, T2 hypointense non-enhancing lesion with capsular retraction in segment VI, consistent with a near-complete response LI-RADS **LR-TR non-viable** (Fig. 1).

MRI features were **indicative of a near-complete response**, with no arterial enhancement, consistent with **nonviable tumour** under LI-RADS Treatment Response (LR-TR) criteria and post-radiation fibrosis. Tumour markers showed a mildly rising trend in Alpha-fetoprotein (AFP) from 12.6 to 35. The patient remains clinically stable with no new complaints.

In this case, focal FAPI avidity within the treated liver segment was ultimately attributed to post-radiation fibrosis on the basis of correlative MRI and stable tumour markers, consistent with **false-positive** uptake.

#### Diagnostic challenge and resolution

The primary diagnostic challenge was differentiating true tumour recurrence from post-treatment inflammatory or fibrotic changes, given the increased FAPI uptake. FAPI avidity can be observed in both malignant processes and benign conditions, such as inflammation and fibrosis. Based on the comprehensive assessment, including the discordant findings between [⁶⁸Ga]Ga-FAPI PET/CT and MRI, the patient’s stable clinical course, and the known biological behaviour of FAPI uptake in fibrotic processes, the increased FAPI avidity was ultimately attributed to post-SBRT fibrosis, mimicking tumour recurrence. This highlights the critical role of multimodal imaging correlation in complex post-treatment scenarios.

#### Outcome

Hepatic segment VI has shown no morphological evidence of local recurrence on serial MRI, consistent with the interpretation of false-positive FAPI uptake secondary to post- SBRT fibrosis. The patient has continued the systemic immunotherapy for over two years, which is well-tolerated and remains ECOG PS-1.

**Table 1:** Timeline of patient’s clinical course and imaging (Case 1). Timeline illustrating key events for Case 1, including diagnosis, SBRT treatments (liver and iliac crest), systemic therapy cycles, and the dates of the MRI and [⁶⁸Ga]Ga-FAPI PET/CT scans

Date	Event
Jan 2024	Metastatic HCC confirmed; AFP 712; FAPI PET/CT: right-lobe liver lesions; left iliac bone metastasis; non-FAPI avid small fissural lung nodule (benign); immunotherapy (atezolizumab + bevacizumab) course 1 initiated.
February 2024	SBRT to left iliac crest (30 Gy/3#) completed.
Apr 2024	FAPI PET/CT shows interval regression in hepatic FAPI uptake and resolution in iliac lesion uptake; partial response
Oct 2024	FAPI PET/CT shows further regression in hepatic lesions; left iliac lesion non-avid; partial response.
November 11, 2024	SBRT to liver (50 Gy/5#) completed.
Mar 3, 2025	FAPI PET/CT shows diffuse increased FAPI avidity in segments V-VI, favored as post-SBRT change; segment V lesion size decreased; small non-FAPI avid segment VII hypodensity; mildly avid peripancreatic node.
June 23, 2025	FAPI PET/CT performed, showing interval increase in FAPI avidity in previously treated liver segment VI.
June 24, 2025	MRI Abdomen performed, showing T1 hyperintense, T2 hypointense non-enhancing lesion in segment VI with capsular retraction, consistent with near-complete response.
December 1, 2025	Patient visit, stable clinical status, AFP mildly rising. Final interpretation of FAPI uptake as post-SBRT fibrosis.

### Case 2

#### Patient information

A 77-year-old gentleman with a background of alcohol-related chronic liver disease (Child-Pugh A6, ALBI Grade 2), type 2 diabetes mellitus, hypertension, and alcohol-associated cognitive impairment presented in February 2023 with a radiologically diagnosed HCC (BCLC Stage A). Cross-sectional imaging (CECT and MRI, February 2023) demonstrated a dominant 2.5 × 2.6 cm arterially enhancing, washout-positive lesion in the right hepatic lobe and a 10 mm lesion in segments VII/VIII, against a background of liver cirrhosis, portal hypertension, and early oesophageal varices. Serum AFP was 16.4 ng/mL at baseline. No liver biopsy was performed; the diagnosis was established on radiological criteria. Following multidisciplinary tumour board discussion (March 21, 2023), the patient was planned for SBRT with curative intent.

#### Treatment

He received SBRT (40 Gy in 5 fractions, VMAT-DIBH technique) from March 20–30, 2023, which was well tolerated with mild constipation.

#### Clinical course and diagnostic assessment

The patient’s clinical course is summarised in Table 2. At six weeks post-SBRT (May 2023), AFP had decreased to 16.8 ng/mL with bilirubin 1.8 mg/dL and albumin 3.4 g/dL, consistent with excellent early response. MRI at three months (July 2023) demonstrated good radiological response with regression of the treated lesions, and surveillance was continued. At seven months post-SBRT (October 2023), the patient developed an episode of hepatic encephalopathy (serum ammonia 145 µmol/L, asterixis positive), managed conservatively on an outpatient basis with anti-encephalopathy measures, with good subsequent recovery.

At eleven months post-SBRT (January 2024), serum AFP had risen to 260 ng/mL. MRI abdomen (January 25–29, 2024) demonstrated an interval reduction in the size and number of hepatic lesions consistent with post-radiation change, without a morphologically defined residual or recurrent lesion. Given the discordance between the rising AFP and the favourable MRI findings, the patient opted for whole-body [⁶⁸Ga]Ga-FAPI PET/CT (January 30, 2024) to evaluate for extrahepatic progression.

**Fig. 2:**
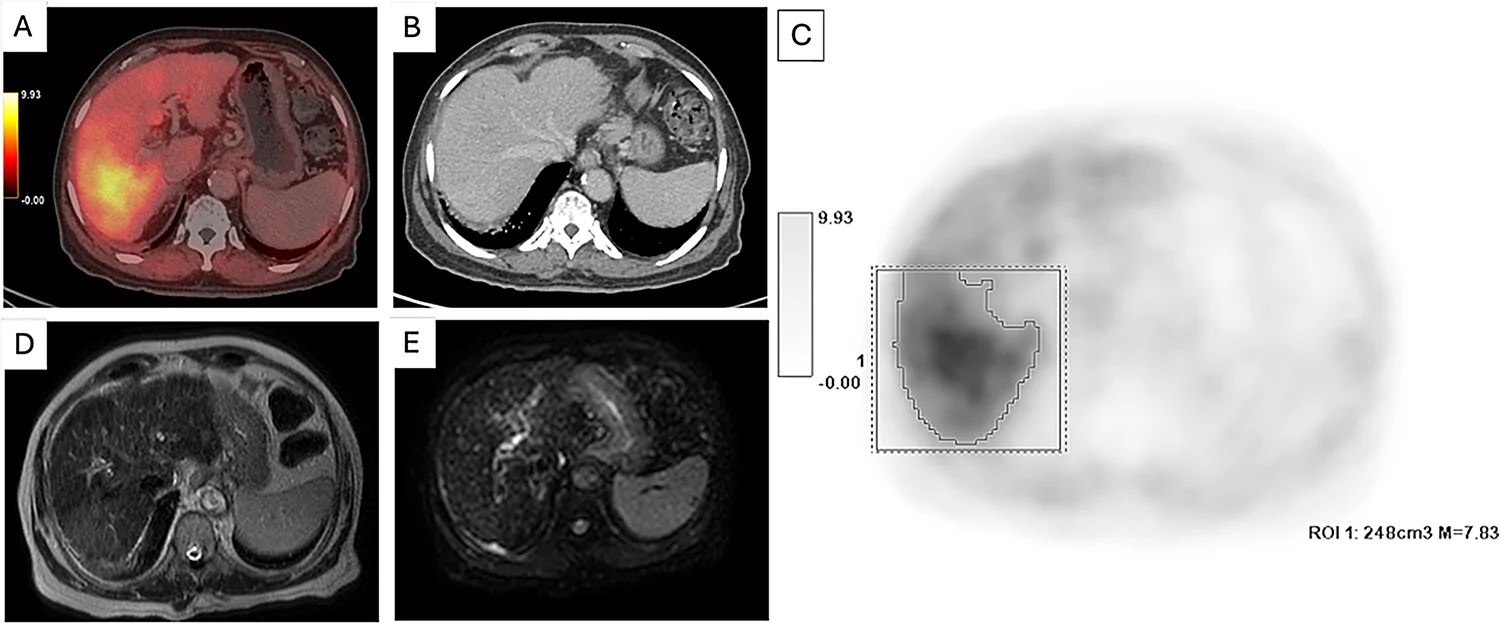
(**A**) Fused Gallium 68 Fibroblast activated protein inhibitor (Ga-68 FAPI) Positron Emission Tomography/Contrast enhanced computed tomography (PET/CECT) images showing diffusely increased FAPI uptake in segment VII and VIII of liver with standardised uptake value maximum of 7.83. (**B**) The corresponding axial section on CECT image shows relatively increased attenuation with no significant enhancement. (**C**) Standalone PET image in grayscale bar showing the maximum standardised uptake value (M = 7.83). (**D**, **E**) Plain MRI shows ill-defined T2/STIR hyperintense area seen in segment VII of liver not showing diffusion restriction. No obvious well-defined lesion identified

[⁶⁸Ga]Ga-FAPI PET/CT demonstrated ill-defined, intensely FAPI-avid lesions in hepatic segments VII/VIII (7.5 × 6 × 5.5 cm, SUVmax 7.83) with mild washout in the delayed phase, corresponding to the previously irradiated field (Fig. 2).

A second, mildly FAPI-avid subcapsular hypodensity (1.2 × 1.4 cm) was noted in segment VIII without significant enhancement. No distant metastases or skeletal lesions were identified.

#### Diagnostic challenge and resolution

The primary diagnostic challenge was the convergence of a rising AFP, an intensely FAPI-avid lesion in the irradiated field with mild delayed washout, and a second indeterminate segment VIII lesion features that could individually raise concern for recurrence or new HCC. However, MRI performed concurrently demonstrated an ill-defined T2/STIR hyperintense signal alteration in segment VII without diffusion restriction and without a discrete morphological lesion, findings inconsistent with viable tumour. The mild delayed washout on [⁶⁸Ga]Ga-FAPI PET/CT, while a feature that can overlap with malignant imaging characteristics, was interpreted in the context of the discordant MRI and the known behaviour of FAPI in post-radiation fibroinflammatory tissue. Multidisciplinary tumour board review (February 1, 2024) concluded that the imaging findings most likely represented post-radiation fibroinflammatory change in the treated field, and recommended medical oncology and palliative medicine follow-up without further intervention.

#### Outcome

At the 14-month post-SBRT review (April 2024), the FAPI uptake was confirmed as a false-positive post-RT change with no recurrence or residual viable disease identified. Based on the available radiological assessment and multidisciplinary consensus at the time of imaging, the FAPI uptake was deemed a false-positive post-SBRT change. It should be noted that the patient’s overall clinical course was complicated by progressive alcohol-related cognitive impairment and declining functional status (ECOG PS-2). The false-positive interpretation therefore remains based on the evidence available at the index evaluation.

**Table 2:** Timeline of patient’s clinical course and imaging (Case 2)

Date	Event
February 2023	Initial diagnosis: HCC BCLC Stage A, 2 lesions; AFP 16.4 ng/mL; CECT and MRI show dominant 2.5 × 2.6 cm arterially enhancing lesion in right hepatic lobe with 10 mm lesion in segments VII/VIII. Background cirrhosis, portal hypertension, early oesophageal varices. Radiological diagnosis (no liver biopsy). Supportive care commenced for alcohol-related cognitive impairment and liver disease.
March 30, 2023	SBRT 40 Gy/5# (VMAT, DIBH technique) to dominant liver lesion; delivered March 20–30, 2023. Well tolerated; mild constipation only. Multidisciplinary tumour board intent: curative.
April 10, 2023	First post-SBRT review: no pain, bloating, or reflux; occasional loss of appetite; sleep disturbances noted by family. Surveillance initiated.
May 8, 2023	Post-SBRT 6 weeks: AFP decreased to 16.8 ng/mL; bilirubin 1.8 mg/dL; albumin 3.4 g/dL. Excellent symptomatic improvement.
July 5, 2023	Post-RT 3 months: MRI abdomen demonstrates good radiological response with regression of treated lesions. Surveillance continued.
October 2023	Hepatic encephalopathy episode: serum ammonia 145 µmol/L, asterixis positive. MRI brain normal. Anti-encephalopathy measures commenced on outpatient basis; patient declined admission. Good subsequent recovery.
January 25–29, 2024	Post-SBRT 11 months: AFP increased to 260 ng/mL. MRI abdomen shows interval reduction in size and number of hepatic lesions consistent with post-radiation change; no morphologically defined residual or recurrent lesion. Whole-body [⁶⁸Ga]Ga-FAPI PET/CT requested to evaluate for extrahepatic progression.
January 30, 2024	[⁶⁸Ga]Ga-FAPI PET/CT: FAPI-avid ill-defined lesion in segments VII/VIII (7.5 × 6 × 5.5 cm, SUVmax 7.83) with mild washout in delayed phase, corresponding to irradiated field; second mildly FAPI-avid subcapsular hypodensity in segment VIII (1.2 × 1.4 cm) without significant enhancement. No distant or skeletal metastases.
February 1, 2024	Multidisciplinary tumour board review: FAPI findings interpreted as likely post-radiation fibroinflammatory change in treated field. Palliative intent confirmed; medical oncology and palliative medicine follow-up advised without further intervention.
April 24, 2024	Long-term follow-up (14 months post-SBRT): ECOG PS-2; memory impairment and walking/speaking difficulties persisting. FAPI uptake confirmed as post-RT fibroinflammatory change; no recurrence or residual viable disease identified. patient managed with supportive care only.

## Discussion

Fibroblast activation protein (FAP) is a type II transmembrane serine protease selectively overexpressed on cancer-associated fibroblasts (CAFs), which is a major component of the tumour microenvironment in HCC (Huang et al. 2022). FAP expression is minimal in normal adult liver parenchyma but is upregulated in activated fibroblasts associated with chronic liver disease, cirrhosis, and hepatic malignancies. This biological profile has led to the development of radiolabelled FAP inhibitors (FAPI) for PET imaging, which enables indirect visualisation of the tumour stroma rather than tumour cells themselves (Huang et al. 2022).

Conventional PET tracers like [^18^F] FDG PET/CT are insensitive for many HCCs, particularly well- or moderately differentiated tumours with high glucose-6-phosphatase activity. Moreover, detection rates of [^18^F] FDG PET in primary HCC are typically < 50% (Wang et al. 2021; Lin et al. 2012). In contrast, FAPI PET/CT exploits a different biological target. Comparative studies show a higher detection of HCC by FAPI than by FDG. In a study by Wang et al., [⁶⁸Ga]Ga-FAPI-04 PET detected 85.7% of HCC lesions versus 57.1% for [^18^F] FDG, a disparity that was especially large in small (≤ 2 cm) and low-grade tumours. The tumour-to-background ratio (TBR) for FAPI was also higher (Wang et al. 2021), suggesting the low normal liver uptake of FAPI. Recent evidence confirms that FAPI PET/CT has markedly higher sensitivity than conventional [^18^F] FDG PET/CT for detecting HCC lesions (Singh et al. 2024). Meta-analyses report pooled sensitivities around 94–98% for FAPI versus roughly 56–63% for FDG in liver tumours, reflecting FAPI’s higher tumour-to-background contrast. In HCC-specific analyses the advantage is even more striking (FAPI ~ 98.5% vs. FDG ~ 60.9%) (Singh et al. 2024). This superior detection stems from the high avidity of HCC stroma for FAP ligands combined with the low physiological liver background uptake of [⁶⁸Ga]Ga-FAPI (Manuppella et al. 2024; Singh et al. 2024).

Other tracers have been explored like [^11^C]acetate PET, which can complement FDG, and novel agents like PSMA-targeted ligands have been tested. Notably, a recent prospective study reported that combining FAPI with [^11^C] acetate PET yielded the highest sensitivity for HCC detection (88.6% of lesions) (Xu et al. 2025), outperforming any single agent. Overall, FAPI PET/CT appears superior to FDG PET/CT in both sensitivity and lesion conspicuity for HCC, while tracers such as [^11^C] acetate or PSMA remain investigational adjuncts.

Despite its sensitivity, FAPI PET/CT is not perfectly tumour specific. Any process that activates fibroblasts, wound healing, fibrosis, or inflammation may yield FAPI uptake (Manuppella et al. 2024). Ablative therapies like transarterial chemoembolization (TACE), radiofrequency ablation (RFA) or stereotactic radiotherapy induce local inflammation and scar formation, which can be seen as increased uptake on FAPI imaging. In one report, Siripongsatian et al. observed two cases of [⁶⁸Ga]Ga-FAPI- 46 uptake at prior TACE sites that MRI showed to be benign post-treatment fibrosis (Siripongsatian et al. 2022). Chen et al. likewise described a wedge-shaped area of [^18^F]F-FAPI uptake around an RFA-treated lesion that proved to be stromal fibrosis rather than tumour (Chen et al. 2022). Inflammatory conditions anywhere (e.g. arthritis, infection, granulomas) also cause false positives (Manuppella et al. 2024). Hence, following locoregional therapy or in benign fibrotic conditions, an increased FAPI signal must be interpreted with caution, as it may reflect healing tissue rather than active cancer (Manuppella et al. 2024).

The two cases presented in this report exemplify these issues. In Case 1, a FAPI avid liver lesion after SBRT was ultimately shown by MRI to represent a fibrotic scar rather than a tumour. This parallels prior observations of post-ablation fibrosis producing linear or wedge-shaped FAPI uptake (Manuppella et al. 2024). Without MRI correlation, such findings could be misread as recurrence. Clinical-radiologic correlation after SBRT is therefore essential. Although a rising serum AFP may raise concern for recurrence, AFP must be interpreted in context. In most SBRT responders, AFP levels decline substantially after treatment. In a recent MRI-based SBRT cohort, patients with elevated baseline AFP demonstrated a rapid reduction (median decline around 96.5% at 3 months), with AFP normalisation achieved during follow-up in the absence of radiologic progression (Mai et al. 2023). Persistent arterial phase hyperenhancement alone does not suggest recurrence. Post-SBRT radiologic response evolves slowly, with APHE frequently persisting for up to 9–12 months despite effective local tumour control. Accordingly, standard response criteria such as mRECIST should be applied cautiously in the early post-treatment period, and treatment efficacy is more reliably assessed at later follow-up, when in-field progression rates are low and true recurrence is better distinguished from expected post-radiation changes (Serafini et al. 2022; Mendiratta-Lala et al. 2019). As highlighted by Mai et al., true local progression after SBRT is defined by lesion growth accompanied by renewed APHE with washout and increased diffusion or T2 signal intensity, whereas persistent or evolving enhancement patterns in the absence of size increase or diffusion restriction should not be interpreted as treatment failure (Mai et al. 2023). In other words, consistent with the findings of Mai et al., in the absence of lesion enlargement or increasing diffusion/T2 signal, active intervention is not recommended despite persistent enhancement patterns; isolated biologic or metabolic abnormalities (such as tracer uptake) without corroborative morphologic progression should be interpreted cautiously (Mai et al. 2023).

These findings reinforce the value of multimodal imaging in HCC surveillance. Structural imaging with contrast-enhanced MRI or CT remains the standard for detecting recurrence and characterising indeterminate lesions. FAPI PET/CT should complement, not replace, conventional imaging. In our cases, MRI was crucial for adjudicating the FAPI findings. Despite extensive study of FAPI PET/CT in oncology, we found no previous reports specifically examining FAPI PET/CT in HCC patients after SBRT. SBRT is an established liver-directed therapy that, like RFA or TACE, induces tumour necrosis followed by fibrosis. Our cases thus represent a novel observation: SBRT-induced hepatic fibrosis can lead to false-positive FAPI uptake. This highlights a previously unreported challenge in interpreting FAPI PET/CT for post-radiation HCC. Given the absence of prior literature on FAPI PET/CT following hepatic SBRT, our report provides new insights and a warning that post-SBRT fibroblast activation may confound FAPI-based imaging. A comparative overview of imaging findings, treatment parameters and final diagnostic interpretation for both the cases is presented in Table 3.

**Table 3:** Imaging metrics, diagnostic interpretation, and SBRT treatment summary for both cases

Case	Lesion location (liver segment)	Prescription (Gy/ fractions)	Biopsy (Y / *N* / specify)	FAPI SUVmax	MRI LR-TR / LI-RADS	Interpretation
Case 1	Segment VI (treated lesion)	50 Gy / 5 fractions.	Biopsy: No liver biopsy. Biopsy from Iliac lesion suggestive of HCC	Increased mean FAPI avidity (SUV Max 8.20)	MR Shows non LR-TR non-viable lesion with adjacent peripheral enhancement shows interval decrease in size post treatment with no increase in adjacent peripheral enhancement.	Interpreted as post-SBRT fibrosis / treatment effect
Case 2	Segments VII/VIII (dominant lesion reported)	40 Gy / 5 fractions.	Biopsy: No liver biopsy; diagnosis based on radiological criteria.	SUVmax 7.83 reported on PET/CT	MRI shows ill defined T2/STIR Hyperintense area not showing diffusion restriction No obvious lesion identified in the present study.	Interpreted as likely post-radiation change.

## Conclusions

In this paired case series, both [^68^Ga]Ga-FAPI PET/CT findings within previously irradiated liver segments were ultimately interpreted as false-positive, based on contemporaneous MRI features of non-viable tissue, the clinical course, and multidisciplinary review. These cases illustrate a key limitation of FAPI imaging in the post-SBRT treatment liver: while highly sensitive for fibroblast-rich lesions and valuable for detection in untreated HCC, FAPI is not tumour-specific and may identify treatment-related fibroinflammatory changes after locoregional therapies (including SBRT). Consequently, isolated FAPI avidity in a treated field should not be taken as definitive evidence of recurrence without concordant anatomical imaging and biochemical trends. To our knowledge, this report is among the first to document FAPI avidity after hepatic SBRT that correlates with MRI proven post-radiation fibrosis, highlighting the need for prospective studies to define the clinical role of FAPI PET/CT in post-treatment surveillance.

## Data Availability

The datasets used and/or analysed during the current study are not publicly available due to patient privacy considerations but are available from the corresponding author on reasonable request.
